# Neuroinflammation mediates noise-induced synaptic imbalance and tinnitus in rodent models

**DOI:** 10.1371/journal.pbio.3000307

**Published:** 2019-06-18

**Authors:** Weihua Wang, Li. S. Zhang, Alexander K. Zinsmaier, Genevieve Patterson, Emily Jean Leptich, Savannah L. Shoemaker, Tatiana A. Yatskievych, Robert Gibboni, Edward Pace, Hao Luo, Jinsheng Zhang, Sungchil Yang, Shaowen Bao

**Affiliations:** 1 Department of Physiology, University of Arizona, Tucson, Arizona, United States of America; 2 Helen Wills Neuroscience Institute, University of California, Berkeley, California, United States of America; 3 Department of Otolaryngology, Wayne State University, Detroit, Michigan, United States of America; 4 Department of Communication Sciences and Disorders, Wayne State University, Detroit, Michigan, United States of America; 5 Department of Biomedical Science, City University of Hong Kong, Kowloon, Hong Kong; Max-Planck-Institut fur experimentelle Medizin, GERMANY

## Abstract

Hearing loss is a major risk factor for tinnitus, hyperacusis, and central auditory processing disorder. Although recent studies indicate that hearing loss causes neuroinflammation in the auditory pathway, the mechanisms underlying hearing loss–related pathologies are still poorly understood. We examined neuroinflammation in the auditory cortex following noise-induced hearing loss (NIHL) and its role in tinnitus in rodent models. Our results indicate that NIHL is associated with elevated expression of proinflammatory cytokines and microglial activation—two defining features of neuroinflammatory responses—in the primary auditory cortex (AI). Genetic knockout of tumor necrosis factor alpha (TNF-α) or pharmacologically blocking TNF-α expression prevented neuroinflammation and ameliorated the behavioral phenotype associated with tinnitus in mice with NIHL. Conversely, infusion of TNF-α into AI resulted in behavioral signs of tinnitus in both wild-type and TNF-α knockout mice with normal hearing. Pharmacological depletion of microglia also prevented tinnitus in mice with NIHL. At the synaptic level, the frequency of miniature excitatory synaptic currents (mEPSCs) increased and that of miniature inhibitory synaptic currents (mIPSCs) decreased in AI pyramidal neurons in animals with NIHL. This excitatory-to-inhibitory synaptic imbalance was completely prevented by pharmacological blockade of TNF-α expression. These results implicate neuroinflammation as a therapeutic target for treating tinnitus and other hearing loss–related disorders.

## Abstract 2

Prolonged exposure to loud noises causes neuronal hyperexcitability and increases the risk of tinnitus. This study reveals that this type of tinnitus is mediated by noise-induced neuroinflammation; blockade of neuroinflammatory responses prevents noise-induced neuronal excitation/inhibition imbalance and tinnitus.

## Introduction

Hearing loss is a widespread condition that affects approximately 500 million individuals [1]. It is associated with tinnitus, hyperacusis, and central auditory processing disorder [2]. The mechanisms by which hearing loss leads to these centrally mediated auditory pathologies are still unknown. Hearing loss can cause a range of cellular and physiological changes in the auditory pathway, including neuronal cell loss, altered synaptic transmission and ion channel function, distorted sensory maps, and abnormal neuronal firing patterns [3–15]. All of these changes have been proposed as potential mechanisms for disorders such as tinnitus [16, 17]. Attempts have been made to treat hearing loss–related tinnitus by targeting specific cellular and physiological changes, but success has been limited [18]. The failure to alleviate tinnitus by targeting individual molecular and cellular mechanisms suggests that tinnitus could be mediated by multiple, parallel mechanisms [3–5, 16, 17]. Blocking only one of the mechanisms is ineffective as the other mechanisms can still lead to tinnitus perception. Consequently, upstream events that link noise-induced hearing loss (NIHL) to the subsequent cellular and physiological changes must be examined in order to develop tinnitus treatments.

Increasing evidence indicates that NIHL and conductive hearing loss can lead to inflammatory responses, such as the activation of microglia and the release of proinflammatory cytokines [19–21], in the early stages of the central auditory pathway. Neuroinflammation is the central nervous system’s response to external and internal insults, such as infection, injury, diseases, and abnormal neural activity [22]. It mobilizes microglia to remove invading pathogens and damaged brain cells through phagocytosis [23, 24]. Microglia can present target antigens from pathogens and damaged brain cells to cytotoxic T cells, which then further attack the targets [25]. Responding to insult, microglia will release proinflammatory cytokines that are involved in neural repair as well as cell death [26]. In addition to interacting with pathogens and injured cells, microglia and their released cytokines modulate the functions of normal neurons. For example, microglia play an important role in neural development, maturation, plasticity, and aging [27–29]. Proinflammatory cytokines also modulate neuronal functions such as synaptic transmission, plasticity, and membrane excitability [13, 30–33].

Although neuroinflammation is important in maintaining homeostasis of the central nervous system against external and internal insults, it can be detrimental if it becomes overactive or chronic [23]. Persistent neuroinflammation is a major pathological component of brain diseases such as autism, schizophrenia, Alzheimer’s disease, Parkinson’s disease, multiple sclerosis, traumatic brain injury, and ischemia [34–38]. Chronic neuroinflammation resulting from noise exposure and hearing loss has been reported in the early stages of the central auditory pathway [19–21]. However, the impact of hearing loss–induced inflammation on neuronal function and its role in tinnitus, hyperacusis, and central auditory processing disorder have not yet been explored. In this study, we characterized neuroinflammation in primary auditory cortex (AI) of mice with NIHL and examined its role in noise-induced electrophysiological and behavioral pathologies. Our results indicate that neuroinflammation plays an essential role in a noise-induced excitatory-to-inhibitory synaptic imbalance and tinnitus in a rodent model.

## Results

### Noise exposure results in elevated expression of proinflammatory cytokines in AI

**Fig 1:**
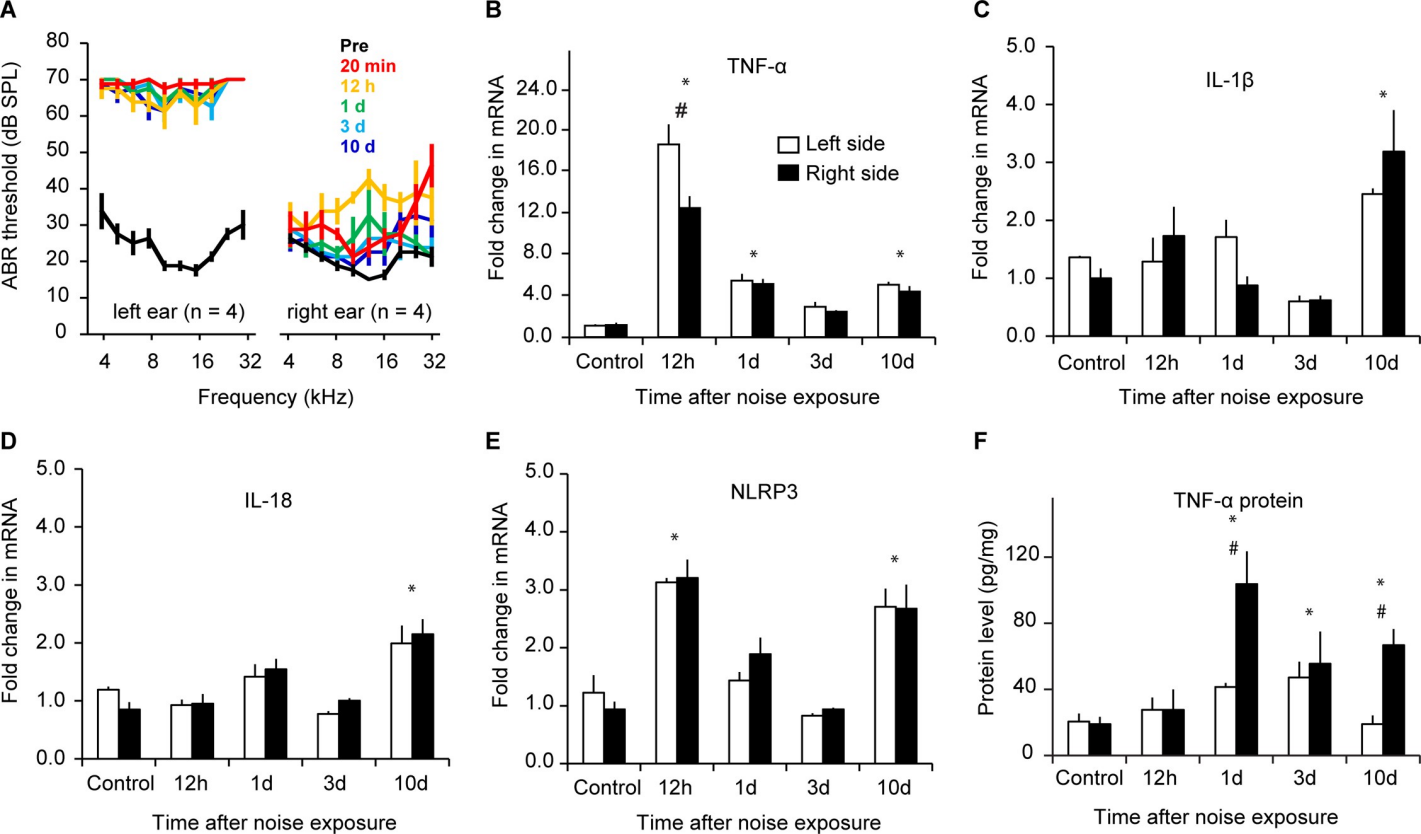
Noise exposure results in elevated expression of proinflammatory cytokines in AI. A. Monaural exposure to loud noises caused long-lasting ABR threshold increases of up to 50 dB in the exposed left ear and temporary threshold increases in the protected right ear that recovered to less than 10 dB in 10 days. B. TNF-α mRNA level increased rapidly within 12 hours of noise exposure, with a stronger ipsilateral than contralateral increase. The increase was also significant at 1 day and 10 days post noise exposure. C–D. IL-1β and IL-18 mRNA levels both increased in bilateral AI 10 days post noise exposure. E. NLRP3 mRNA level showed biphasic increases at 12 hours and 10 days post noise exposure. F. TNF-α protein levels increased in AI of the right hemisphere 1 day after noise exposure, and the increase persisted to at least 10 days post exposure. Data associated with this figure can be found in S1 Data. Error bars represent SEM. * depicts *P* < 0.01 compared to control; # indicates *P* < 0.01 comparing left and right hemispheres. *n* = 4 mice for each time point in B–F. ABR, auditory brainstem response; AI, primary auditory cortex; IL, interleukin; NLRP3, nod-like receptor protein 3; TNF-α, tumor necrosis factor alpha.

Tumor necrosis factor alpha (TNF-α) expression is elevated by loud noise exposure in the auditory periphery and cochlear nucleus [19–21, 39] and is implicated in tinnitus [40, 41]. We exposed the left ear of C57BL/6 mice to a continuous 8-kHz tone at 112–114 dB sound pressure level (SPL) for 2 hours. After 20 minutes of sound exposure, the exposed ear often lost auditory brainstem responses (ABRs) to the test frequencies at 70 dB, the loudest test level, whereas the protected ear showed approximately 10-dB threshold increases at most of the test frequencies (Fig 1A, *n* = 4 mice for each time point). Two hours of sound exposure resulted in long-lasting threshold shifts of up to 50 dB to the left ear, and weaker, temporary threshold increases in the right ear that recovered to less than 10 dB within 10 days. We then examined the expression of proinflammatory cytokines in AI of mice with NIHL 0.5, 1, 3, and 10 days post noise exposure (*n* = 4 mice for each time point). Real-time-PCR assays showed that the TNF-α mRNA level increased after noise exposure, with stronger increases in the left side (ipsilateral to the exposed ear) (Fig 1B; main effect on exposure, *F*_4,47_ = 43.509, *P* < 0.001; bilateral difference, *F*_1,47_ = 4.530, *P* = 0.039; interaction, *F*_4,47_ = 2.198, *P* = 0.084). The increase in TNF-α transcription peaked at 12 hours after the noise exposure (multiple comparison *P* < 0.001 for 12 hours versus control). Although the level declined over the ensuing days, it was still significantly higher than control at 1 day (Fig 1B, *P* < 0.001) and 10 days (*P* < 0.001) after noise exposure. In contrast to the rapid increase of the TNF-α mRNA level, the expression of proinflammatory cytokines interleukin 1β (IL-1β) and interleukin 18 (IL-18) were significantly up-regulated bilaterally but only 10 days after noise exposure (Fig 1C and 1D; main effect on exposure: IL-1β, *F*_4,27_ = 11.98, *P* < 0.001; IL-18, *F*_4,28_ = 12.42, *P* < 0.001). There were no significant differences in the expression of these two proinflammatory cytokines between the left and right sides of AI (bilateral difference: IL-1β, *F*_1,27_ = 0.000, *P* = 0.999; IL-18, *F*_1,28_ = 0.099, *P* = 0.755; bilateral × exposure interaction: IL-1β, *F*_4,27_ = 1.909, *P* = 0.138; IL-18, *F*_4,28_ = 0.637, *P* = 0.640).

Nod-like receptor protein 3 (NLRP3) is a main structural component of the inflammasome, a multiprotein complex responsible for the activation of inflammatory responses [42]. Monaural noise exposure significantly elevated the expression of NLRP3 12 hours and 10 days after noise exposure (Fig 1E; main effect on exposure, *F*_4,30_ = 26.76, *P* < 0.001). The increase was bilateral, and there were no differences between the two sides (bilateral difference, *F*_1,30_ = 0.134, *P* = 0.717; time × side interaction, *F*_4,30_ = 0.480, *P* = 0.750).

In contrast to the rapidly peaking TNF-α mRNA level, the TNF-α protein level was not significantly altered until 1 day after noise exposure, and the increase was more pronounced in AI of the right hemisphere than the left hemisphere (Fig 1F; main effect on exposure, *F*_4,30_ = 7.015, *P* < 0.001; bilateral difference, *F*_1,30_ = 11.133, *P* = 0.002; interaction, *F*_4,30_ = 3.562, *P* = 0.017; posthoc comparison to the control level, *P* < 0.05 for 1, 3, and 10 days). While the increase in the TNF-α protein level persisted in the right side, it decayed back to the baseline level in the left side by 10 days after the exposure (day 10 compared to corresponding controls: left, *P* > 0.5; right, *P* < 0.05).

### Noise exposure causes microglial deramification in AI

**Fig 2:**
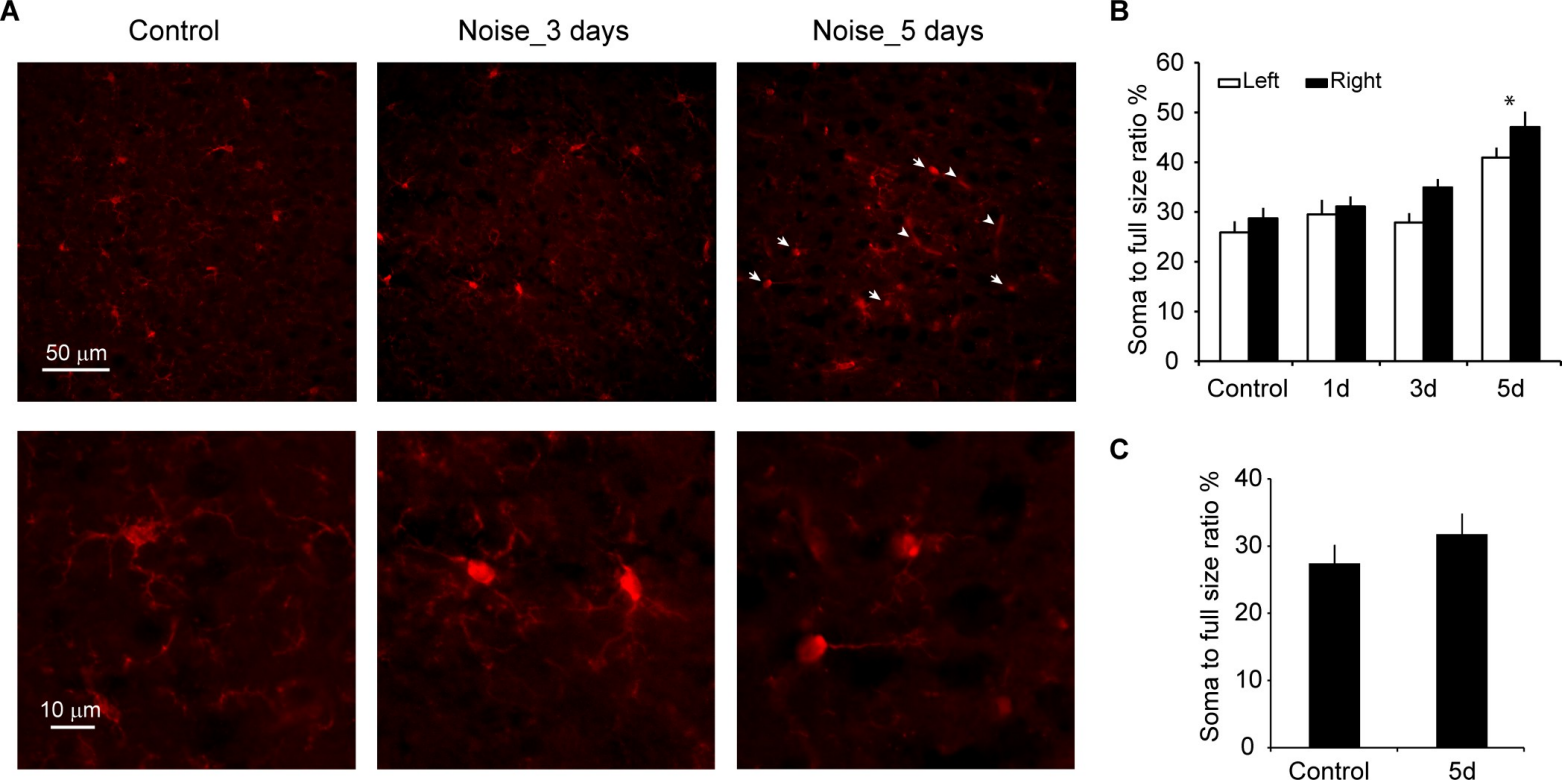
Microglial deramification in AI after noise exposure. A. Representative images of IBA1 antibody–stained microglia in AI of control and noise-exposed mice. Microglia in control mice showed ramified morphology, indicative of their resting state. A proportion of the microglia became activated and transitioned into nonramified and amoeboid shapes (arrows and arrowheads) 5 days after noise exposure. Lower panels show morphologies of ramified (control and Noise_3 days) and deramified (Noise_5 days) microglia. B. The soma-to-whole cell size ratio of microglia was used to measure microglial morphological change as an index of microglial activation. There was a significant increase in the microglial activation 5 days after noise exposure, and the activation was stronger for the right than left side. *n* ≥ 8 for each group. C. No significant increase in microglial activation index was observed in the right visual cortex 5 days after noise exposure. *n* = 17 for control and 22 for 5-day groups. Data associated with this figure can be found in S1 Data. Error bars represent SEM. * depicts *P* < 0.01 comparing left and right sides. AI, primary auditory cortex; IBA1, ionized calcium binding adaptor molecule 1.

During neuroinflammatory responses, microglia are activated and change their morphology from the ramified shape to a nonramified, amoeboid shape [43, 44]. We stained ionized calcium binding adaptor molecule 1 (IBA1), a common microglial marker [45, 46], to examine the microglial morphology in AI. While most of the microglia in the control group had the ramified shape with extended processes, many microglia in the 5-day post noise–exposure group were nonramified or amoeboid (Fig 2A). We quantified this morphological change with an index of the soma-to-whole cell size ratio [47, 48]. A higher soma-to-whole cell size ratio indicates activated (nonramified and amoeboid) microglia [47, 48]. For each group, eight or more sections were collected from both rostral and caudal (higher- and lower-frequency) zones of AI from four mice. The ratio increased significantly for AI microglia 5 days after noise exposure, and the increase was more pronounced in the right hemisphere contralateral to the exposed (left) ear (Fig 2B; sides × days two-way ANOVA: main effect on bilateral difference, *F*_1,68_ = 5.100, *P* = 0.027; main effect on days after exposure, *F*_3,68_ = 17.908, *P* < 0.001; pairwise comparison, day 5 versus control, *P* < 0.01 for both left and right sides). The noise-induced deramification was limited to the auditory cortex and was not observed in the neighboring visual cortex (soma-to-whole cell size ratio in right visual cortex: control 27.2 ± 1.9, noise-exposed 31.7 ± 2.2, ANOVA *F*_1,37_ = 2.244, *P* = 0.143). Taken together, our results so far indicate that monaural exposure to loud noises results in more pronounced neuroinflammation in the contralateral AI, as indicated by increased expression of proinflammatory cytokines and activation of microglia.

### TNF-α knockout mice do not exhibit noise-induced microglial deramification

**Fig 3:**
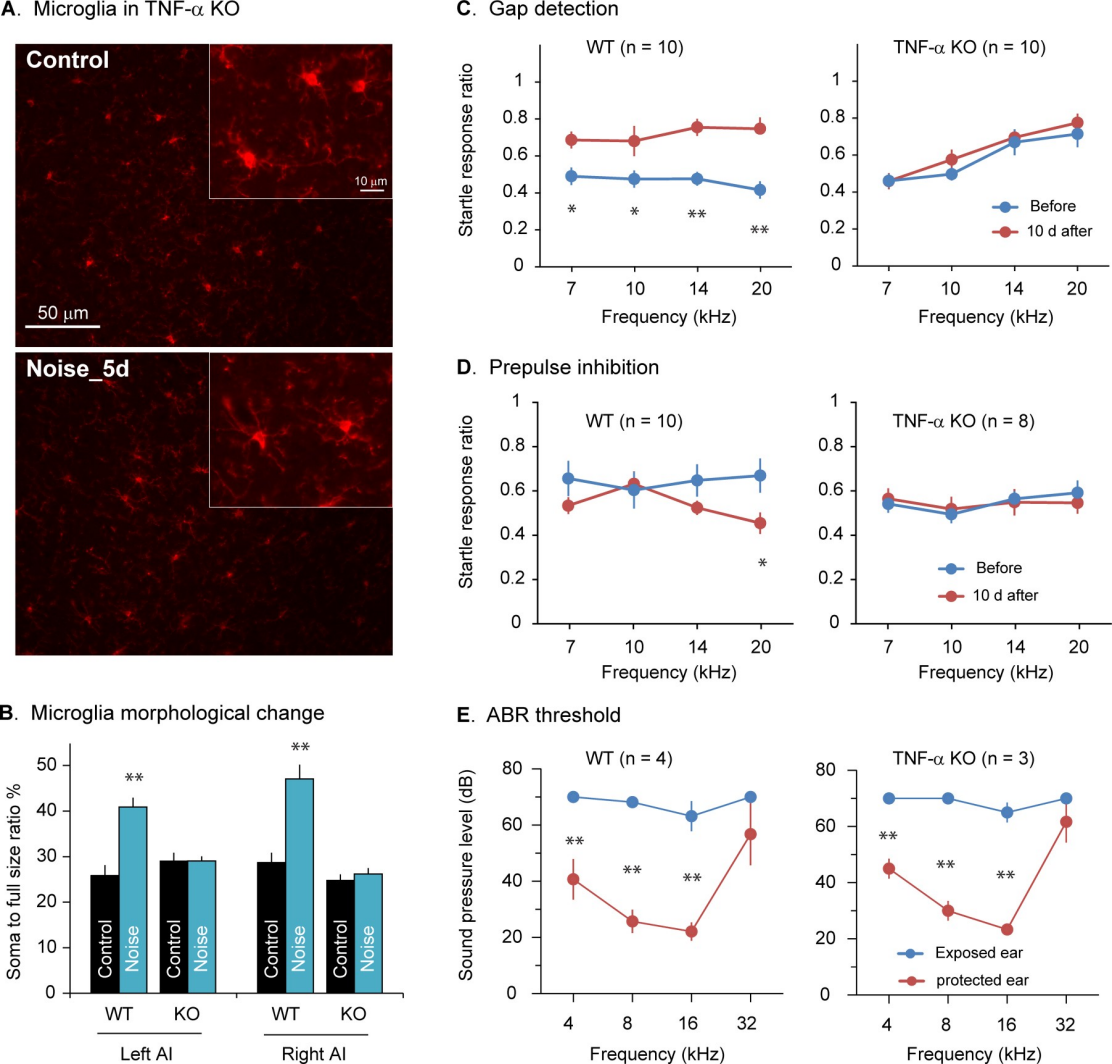
TNF-α KO mice do not show microglial deramification or tinnitus after noise exposure. A. Example images of microglia stained with IBA1 antibody in TNF-α KO mice with (Noise_5d, 5 days after noise exposure) or without noise exposure (Control). Inserts show enlarged images of microglial morphology. B. Noise-induced microglial activation 5 days after noise exposure, as quantified with the soma-to-whole cell size ratio, was absent in TNF-α KO mice. *n* ≥ 12 for each group. C–D. Wild-type but not TNF-α KO mice showed noise-induced tinnitus. Tinnitus and hearing were assessed with the gap detection and PPI, respectively. While gap detection was impaired in wild-type mice 10 days after noise exposure, their PPI was improved at a certain frequency. Neither gap detection nor PPI was altered in TNF-α KO mice by noise exposure. E. Wild-type and TNF-α KO mice showed similar levels of noise-induced increases in ABR thresholds. Data associated with this figure can be found in S1 Data. Error bars represent SEM. * and ** depict *P* < 0.05 and *P* < 0.01, respectively. ABR, auditory brainstem response; IBA1, ionized calcium binding adaptor molecule 1; KO, knockout; PPI, prepulse inhibition; TNF-α, tumor necrosis factor alpha.

TNF-α is a central organizer of neuroinflammatory responses [49–51]. Our findings of the rapid increase in TNF-α expression after noise exposure (Fig 1) and the delayed microglial deramification days later (Fig 2) suggest that TNF-α may play a role in microglial activation. We compared microglial deramification in wild-type and TNF-α knockout mice. We found that while microglia in wild-type mice became less ramified after noise exposure, the microglia in AI in the TNF-α knockout mice remained ramified 5 days after noise exposure (Fig 3A and 3B; genotype × exposure two-way ANOVA: left side, main effect on genotype, *F*_1,59_ = 6.551, *P* = 0.013; exposure effect, *P* < 0.001 for the wild-type group, *P* > 0.5 for the knockout group; right side, effect on genotype, *F*_1,61_ = 30.352, *P* < 0.001; exposure effect, *P* < 0.001 for the wild-type group, *P* > 0.05 for the knockout group). These results indicate that TNF-α knockout mice do not develop the type of neuroinflammatory response that we have observed in AI of wild-type mice after noise exposure.

### Wild-type but not TNF-α knockout mice display evidence of noise-induced tinnitus

Hearing loss is a major risk factor for tinnitus [2]. NIHL has been shown to correlate with behavioral evidence of tinnitus in rodent models [52]. To examine a potential role of noise-induced neuroinflammation in tinnitus, we tested noise-induced tinnitus in TNF-α knockout mice using the acoustic startle reflex-based gap detection test [53]. Gap detection performance was significantly impaired in wild-type mice 10 days after noise exposure compared to before noise exposure, which is considered behavioral evidence of tinnitus (Fig 3C; repeated measures ANOVA, main effect of noise exposure: *F*_1,36_ = 65.366, *P* < 0.001; pairwise comparison: *P* < 0.05 for all four frequencies tested). By contrast, noise exposure did not change gap detection performance in TNF-α knockout mice (*F*_1,36_ = 2.034, *P* = 0.162). A direct comparison of the gap detection performance after noise exposure indicates that it was significantly worse in wild-type mice than in TNF-α knockout mice (*F*_1,72_ = 6.385, *P* = 0.014). We also examined the prepulse inhibition (PPI) of the startle response as a measure of how well the mice could hear the tones used in the gap detection test and to determine if hearing loss contributed to the observed impairment in gap detection. While PPI performance was not changed by noise exposure in the TNF-α knockout mice (Fig 3D; *F*_1,28_ = 0.007, *P* = 0.932), it was significantly improved in the wild-type mice after noise exposure (*F*_1,36_ = 6.172, *P* = 0.018). The lack of impairment in PPI performance in the control mice with monaural NIHL suggests that they could still detect the tones with the protected ear and that acoustic modulation of startle response is still intact in those mice. Furthermore, wild-type and TNF-α knockout mice showed similar ABR thresholds measured after the completion of the behavioral tests (Fig 3E). Together, these results suggest that the proinflammatory cytokine TNF-α is required for the noise-induced tinnitus that we observed 10 days after noise exposure.

### TNF-α infusion in AI results in tinnitus in normal-hearing wild-type and TNF-α knockout mice

**Fig 4:**
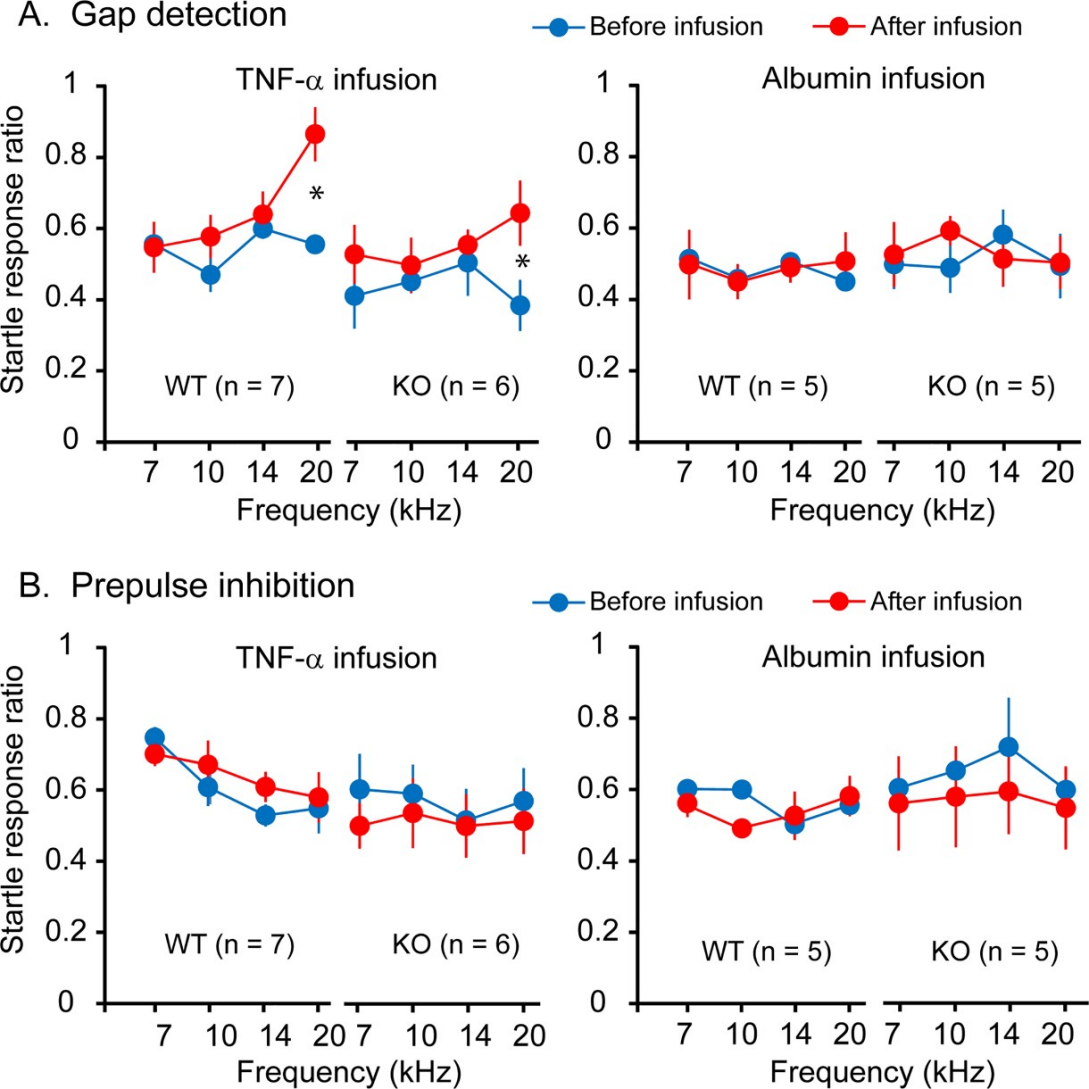
TNF-α infusion in AI results in tinnitus in normal-hearing wild-type and TNF-α KO mice. A. Behavioral evidence of tinnitus was assessed with gap detection. B. The ability to hear tones was assessed with PPI. TNF-α infusion resulted in impaired gap detection in both wild-type and TNF-α KO mice but did not alter their performances in PPI. Albumin infusion did not affect gap detection or PPI in either wild-type or TNF-α KO mice. All mice had normal hearing. Data associated with this figure can be found in S1 Data. All results are presented as mean ± SEM and * indicates *P* < 0.05. AI, primary auditory cortex; KO, knockout; PPI, prepulse inhibition; TNF-α, tumor necrosis factor alpha.

To determine whether the absence of noise-induced tinnitus in TNF-α knockout mice was due to the lack of TNF-α or secondary developmental abnormalities, we infused recombinant TNF-α into the right AI of normal-hearing wild-type and TNF-α knockout mice. After 3 days of recovery, the infused animals underwent gap detection and PPI tests. Our results indicate that TNF-α infusion resulted in impaired gap detection at the frequency of 20 kHz in wild-type mice (Fig 4A; two-way ANOVA, effect of TNF-α infusion, *F*_1,48_ = 9.426, *P* = 0.004) and TNF-α knockout mice (*F*_1,40_ = 5.734, *P* = 0.021). Infusion of albumin did not have the same effect (wild-type: *F*_1,32_ = 0.000, *P* = 0.998; knockout: *F*_1,32_ = 0.135, *P* = 0.716). Furthermore, the PPI performance was not significantly altered in wild-type or knockout mice by either recombinant TNF-α or albumin (Fig 4B; wild-type: recombinant TNF-α, *F*_1,48_ = 0.863, *P* = 0.358; albumin, *F*_1,32_ = 0.887, *P* = 0.356; knockout: TNF-α, *F*_1,40_ = 0.990, *P* = 0.326; albumin, *F*_1,32_ = 1.391, *P* = 0.247). These results suggest that the absence of noise-induced tinnitus in TNF-α knockout mice was due to the lack of TNF-α but not secondary developmental effects.

### Microglial depletion down-regulates TNF-α expression and prevents noise-induced tinnitus

**Fig 5:**
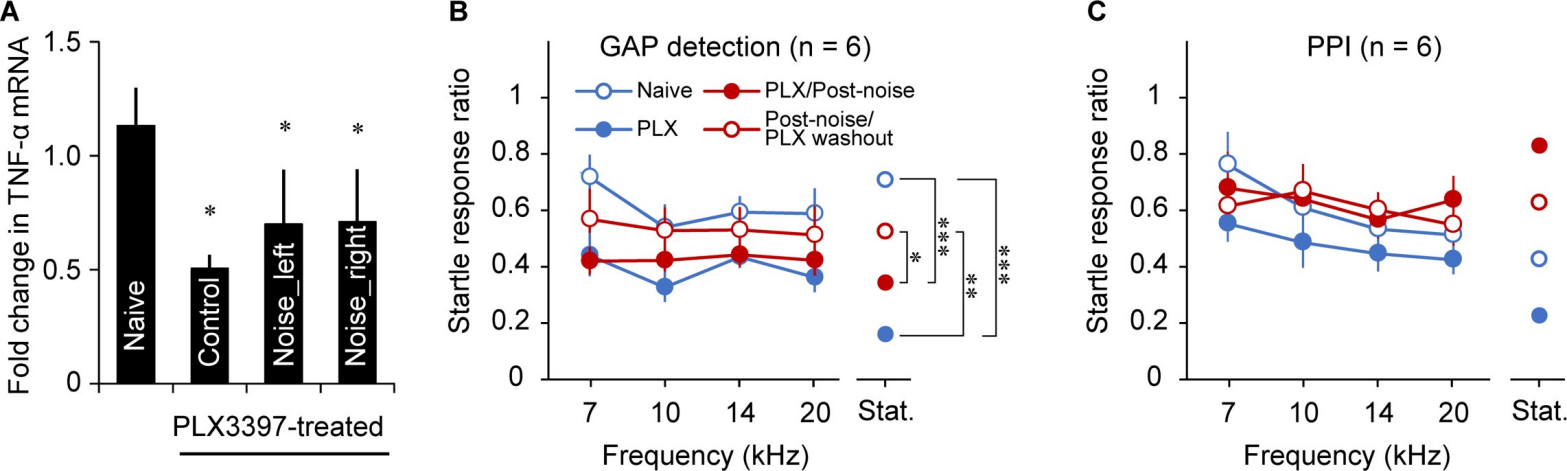
Microglial depletion down-regulates TNF-α expression and prevents noise-induced tinnitus. A. Treatment with PLX3397 suppressed the TNF-α mRNA level in AI and prevented noise-induced increases in TNF-α expression 12 hours after noise exposure. Control mice (*n* = 4) that had undergone PLX3397 treatment and sham exposure had a lower TNF-α mRNA level compared to naïve mice (*n* = 7). In PLX 3397-treated mice (*n* = 4), noise exposure did not significantly increase TNF-α expression in AI of either hemisphere. * indicates *P* < 0.05 compared to naïve. B. Behavioral evidence of tinnitus was evaluated with gap detection. Behavioral tests were conducted at four time points: 1) before PLX 3397 treatment (naïve), 2) after 21 days of PLX3397 treatment (PLX), 3) after noise exposure and still with PLX 3397 treatment (PLX/post noise), and 4) after 7 days of washout of PLX3397 (post noise/PLX washout). Microglial depletion improved basal-level gap detection and prevented noise exposure–induced impairment in gap detection as observed in wild-type mice in Fig 3C. *, **, and *** indicate *P* < 0.05, *P* < 0.01, and *P* < 0.001, respectively. C. Animals’ ability to hear tones, which was measured with PPI, was not altered by PLX treatment or noise exposure (treatment effect, *P* = 0.15). Data associated with this figure can be found in S1 Data. Error bars represent SEM. PPI, prepulse inhibition; TNF-α, tumor necrosis factor alpha.

Both microglia and neurons express TNF-α [21, 32, 54]. To determine the contribution of microglia to noise-induced TNF-α expression and tinnitus, we eliminated a proportion of microglia with PLX3397, an inhibitor of colony-stimulating factor 1 receptor (CSF1R). Administration of PLX3397 for 21 days has been shown to deplete most of the microglia, which recover completely within 7 days of drug washout [55–58]. We found that intraperitoneal (IP) injection of PLX3397 for 21 days significantly reduced baseline TNF-α mRNA level (Fig 5A; naïve versus PLX3397-treated control, *P* < 0.05; *n* = 7 for naïve and *n* = 4 mice for PLX3397-treated control group) and prevented an increase in TNF-α expression 12 hours after noise exposure (Fig 5A; naïve versus PLX3397-treated/noise-exposed groups, *P* < 0.05; PLX3397-treated/control versus PLX3397-treated/noise-exposed groups, *P* = 0.42; *n* = 4 for PLX3397-treated/noise-exposed groups). These results suggest that microglia are activated by noise exposure and contribute to the increase in TNF-α expression.

To examine the effects of PLX3397 on noise-induced tinnitus, we performed noise exposure in a group of six mice that had been treated with PLX3397 for 21 days. Following the noise exposure, PLX3397 was given to the mice for another 10 days. Gap detection and PPI performances were examined at four stages: 1) before administration of PLX3397, 2) on the 21st day of the drug administration, 3) on the 10th day after noise exposure, and 4) 7 days after the withdrawal of PLX3397. Behavioral tests indicated that treatment with PLX3397 improved gap detection performance and prevented noise-induced tinnitus (Fig 5B and 5C). A repeated measures ANOVA revealed a significant treatment effect amongst groups (*F*_3,20_ = 5.115, *P* = 0.009 amongst naïve, PLX [PLX3397], PLX/post noise, and post noise/PLX washout) without a treatment × frequency interaction (*F*_3,20_ = 0.395, *P* = 0.76). Pairwise comparisons indicated that gap detection was significantly improved by PLX3397 administration (*P* < 0.001). Noise exposure did not significantly alter gap detection performance in animals that had received PLX3397 (*P* = 0.33). Furthermore, after the washout of PLX3397 for 7 days, gap detection performance returned to the baseline level before PLX3397 administration (*P* = 0.005 comparing performances before and after washout and *P* = 0.24 comparing performances between naïve and after washout). The treatments had no significant effects on performance in the PPI test (treatment effect, *F*_3,20_ = 1.968, *P* = 0.15; treatment × frequency interaction, *F*_3,20_ = 0.507, *P* = 0.68), suggesting that sound detection and startle performance were not altered by PLX3397 treatment or noise exposure. These results indicate that microglia contribute to noise-induced increase in TNF-α expression and tinnitus.

### Pharmacologically blocking TNF-α expression prevents noise-induced neuroinflammation and tinnitus

**Fig 6:**
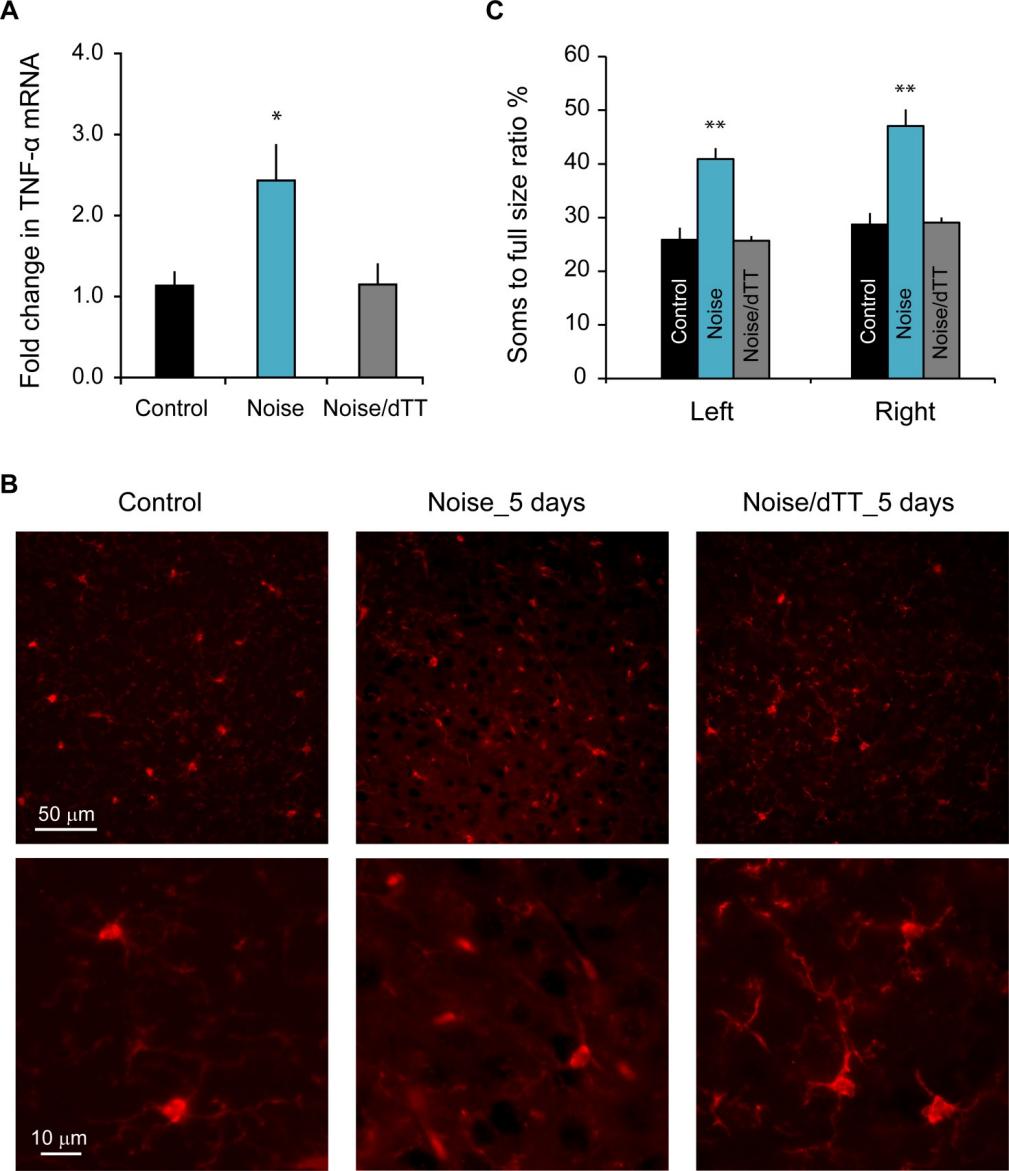
Treatment with dTT prevents noise-induced TNF-α expression and microglial deramification 5 days after the exposure. A. Treatment with dTT completely prevented TNF-α mRNA increase in AI area 5 days after noise exposure. Mice in the Noise group were exposed to noise. Mice in the Noise/dTT group underwent noise exposure followed by 5 days of dTT treatment. *n* = 7 mice for control, 6 for noise, and 4 for noise/dTT groups. B. Example images of microglia in AI visualized with IBA1 antibody staining. C. Noise exposure–induced microglial activation, as quantified with the soma-to-whole cell size ratio, was completely blocked by dTT treatment. Error bars represent SEM. *n* = 12 sections for control, 16 for noise, and 24 for noise/dTT groups. Data associated with this figure can be found in S1 Data. * depicts *P* < 0.05 and ** indicates *P* < 0.01 compared to control. AI, primary auditory cortex; dTT, 3,6′-dithiothalidomide; IBA1, ionized calcium binding adaptor molecule 1; TNF-α, tumor necrosis factor alpha.

**Fig 7:**
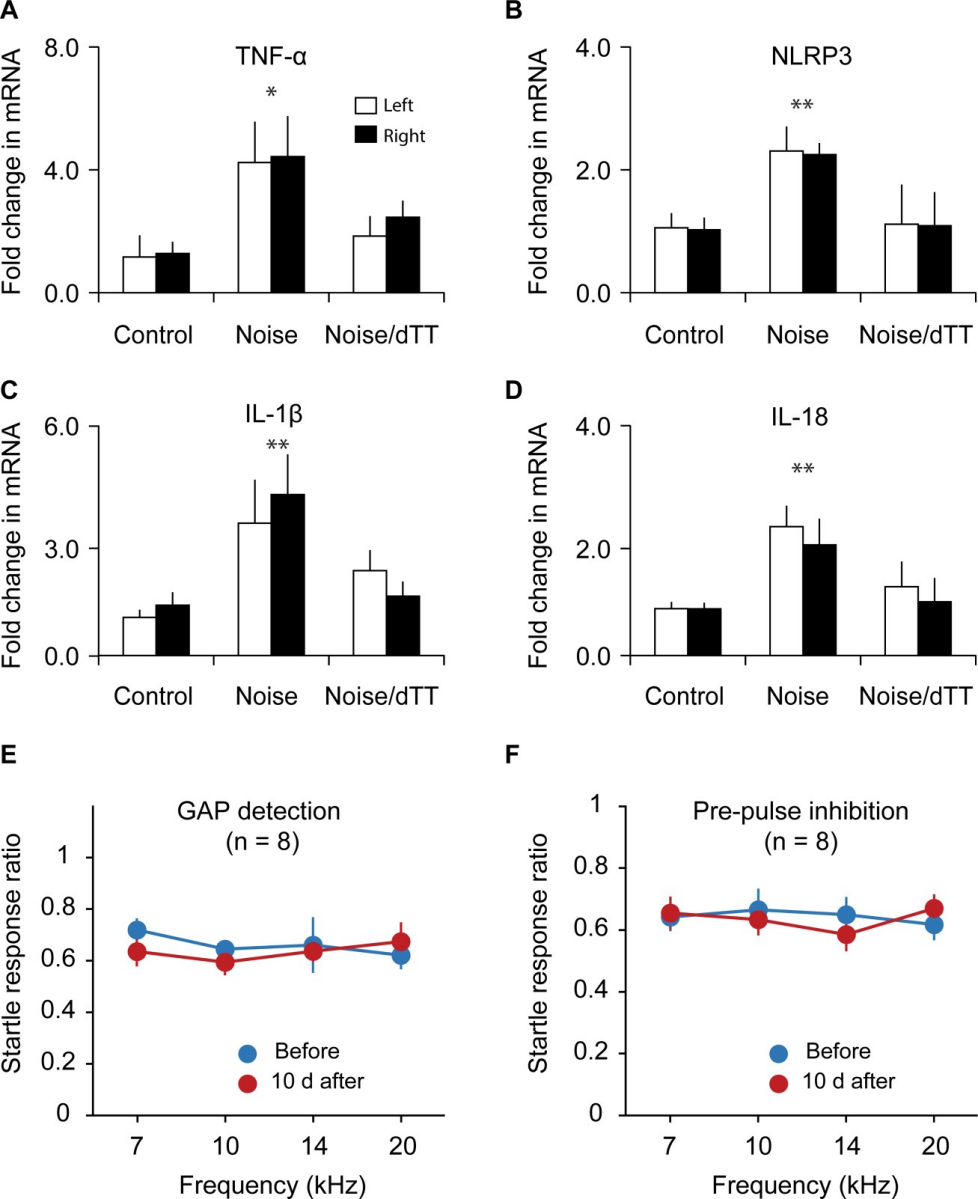
Treatment with dTT prevents noise-induced proinflammatory cytokine expression and tinnitus 10 days after the exposure. A–D. mRNA levels of proinflammatory proteins TNF-α, NLRP3, IL-1β, and IL-18 in AI were up-regulated 10 days after noise exposure, and the up-regulations were completely blocked by dTT treatment. *n* ≥ 4 mice for each group. E. Treatment with dTT completely prevented noise exposure–induced tinnitus, as assessed with gap detection. F. The ability to detect tones, as assessed with PPI, was not altered by noise exposure and dTT treatment. Data associated with this figure can be found in S1 Data. Error bars represent SEM. * depicts *P* < 0.05 and ** indicates *P* < 0.01. AI, primary auditory cortex; dTT, 3,6′-dithiothalidomide; IL, interleukin; NLRP3, nod-like receptor protein 3; PPI, prepulse inhibition; TNF-α, tumor necrosis factor alpha.

To further investigate the role of neuroinflammation in tinnitus, we examined the effect of 3,6′-dithiothalidomide (dTT)—a TNF-α inhibitor that has been shown to reduce neuroinflammation—on noise-induced tinnitus. We treated mice with five daily injections of dTT starting within 1 hour after noise exposure was finished and found that dTT prevented the increase in TNF-α expression (Fig 6A; *F*_2,14_ = 5.75, *P* < 0.05) and microglial morphological changes in AI 5 days after noise exposure (Fig 6B and 6C; left side: *F*_2,45_ = 28.22, *P* < 0.001; right side: *F*_2,49_ = 25.92, *P* < 0.001; *n* = 12 for control, 16 for noise, and 24 for noise/dTT groups). A similar dTT administration regime also completely prevented increases in the proinflammatory cytokines TNF-α, IL-1β, IL-18, and the inflammasome protein NLRP3 10 days after noise exposure (Fig 7A–7D; TNF-α: *F*_2,23_ = 7.91, *P* = 0.002, noise/dTT versus control, *P* = 0.32; NLRP3: *F*_2,22_ = 6.57, *P* = 0.006, noise/dTT versus control, *P* = 0.99; IL-1β: *F*_2,22_ = 13.65, *P* < 0.001, noise/dTT versus control, *P* = 0.20; IL-18: *F*_2,21_ = 8.03, *P* = 0.003; noise/dTT versus control, *P* = 0.68; 4–7 mice were included for each group). These results indicate that dTT administration after noise exposure prevents noise-induced increases in proinflammatory cytokines and microglial activation—two defining characteristics of neuroinflammation.

To examine the effects of dTT on noise-induced tinnitus, we treated mice with daily dTT administration for 5 days starting within 1 hour after the noise exposure. Gap detection and PPI performances were examined before and 10 days after the noise exposure. No significant differences were observed in either gap detection (Fig 7E; repeated measure ANOVA, main effect of noise exposure, *F*_1,30_ = 3.078, *P* = 0.09; noise exposure × frequency interaction, *F*_1,30_ = 2.446, *P* = 0.13) or PPI (Fig 7F; repeated measure ANOVA, main of noise exposure, *F*_1,30_ = 0.340, *P* = 0.56; noise exposure × frequency interaction, *F*_1,30_ = 0.279, *P* = 0.60). Furthermore, these dTT-treated and noise-exposed mice had elevated ABR thresholds that were not different from the thresholds of mice that received noise exposure without dTT treatment (ANOVA, *F*_1,32_ = 2.636, *P* = 0.114). Taken together, these results indicate that pharmacologically inhibiting neuroinflammation with dTT prevents noise-induced tinnitus.

### Treatment with dTT prevents noise-induced excitatory-to-inhibitory synaptic imbalance

**Fig 8:**
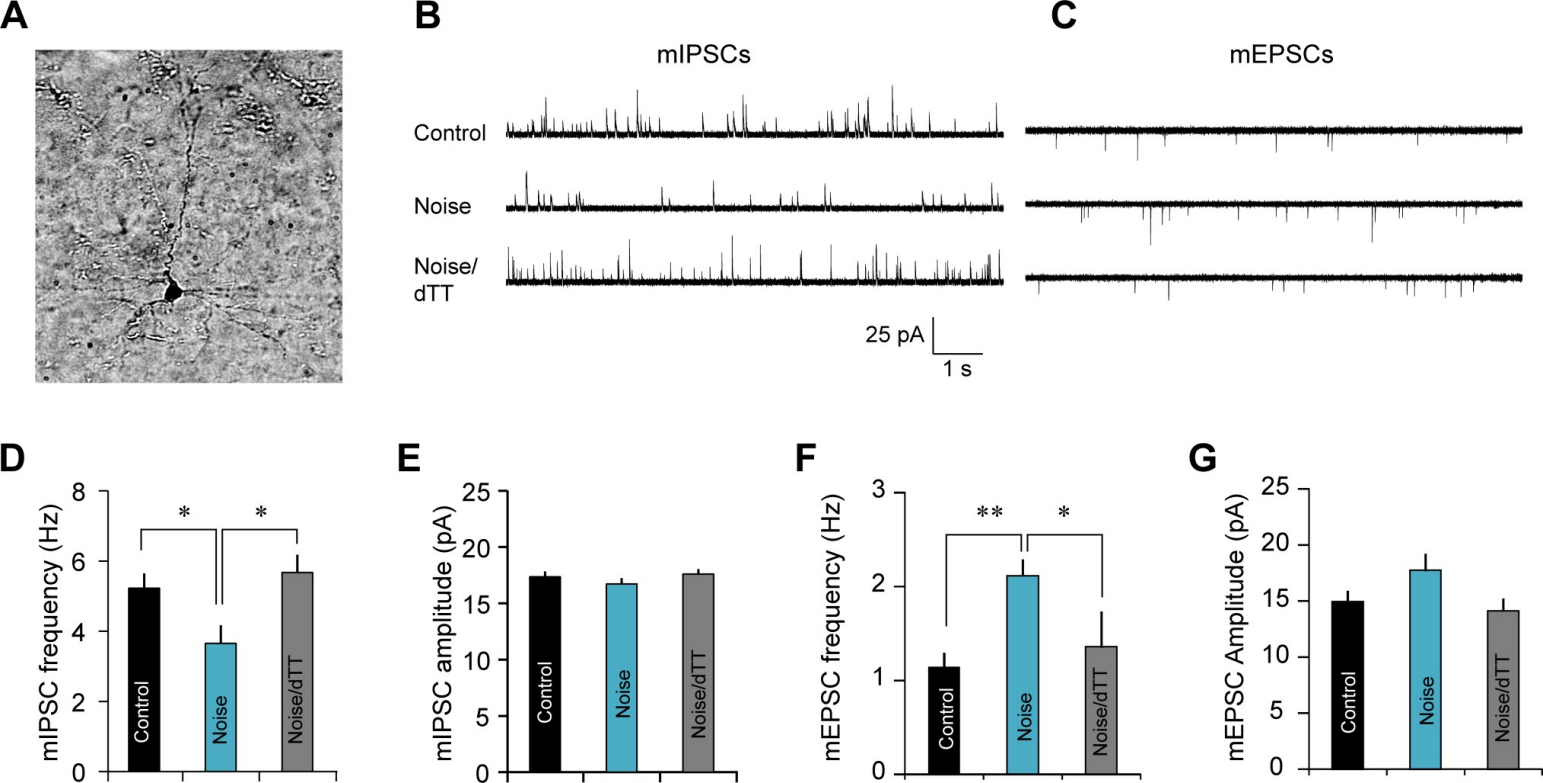
Treatment with dTT prevents noise-induced excitatory-to-inhibitory synaptic imbalance. A. An image of a recorded pyramidal neuron. B–C. Example traces of mIPSCs and mEPSCs recorded from pyramidal neurons from control, noise exposed or noise-exposed and dTT-treated animals. D–G. Frequency and amplitude of mIPSCs and mEPSCs. Noise exposure resulted in a reduction of mIPSC frequency and an increase of mEPSC frequency, both of which were completely blocked by dTT treatment. Error bars depict SEM. *n* = 16, 10, and 19 for mIPSC of the control, noise, and noise/dTT groups and *n* = 6, 9, and 5 for mEPSC of the control, noise, and noise/dTT groups. Data associated with this figure can be found in S1 Data. * depicts *P* < 0.05 and ** indicates *P* < 0.01. dTT, 3,6′-dithiothalidomide; mEPSC, miniature excitatory synaptic current; mIPSC, miniature inhibitory synaptic current.

TNF-α has been shown to change excitatory and inhibitory synaptic functions [13, 59–65]. To determine the role of TNF-α in noise-induced excitatory-to-inhibitory synaptic imbalance, we examined miniature excitatory synaptic currents (mEPSCs) and miniature inhibitory synaptic currents (mIPSCs) in AI pyramidal neurons (Fig 8A) in mice that had undergone noise exposure followed by either dTT or vehicle treatment. We found that the mIPSC frequency was significantly lower in the noise-exposed and vehicle-treated group (*n* = 10) when compared to the sham-exposed control group (*n* = 16) (Fig 8B and 8D; one-way ANOVA, *F*_2,42_ = 3.74, *P* = 0.032; posthoc comparison noise versus control, *P* < 0.05), and this decrease was completely reversed by dTT treatment (*n* = 19) (noise/dTT versus noise, *P* < 0.05; noise/dTT versus control, *P* = 0.49). The corresponding mIPSC amplitudes of the three groups were not significantly different (Fig 8B and 8E; *F*_2,44_ = 0.83, *P* = 0.442). The frequency of mEPSCs increased in the noise-exposed and vehicle-treated group (*n* = 6) compared to the sham-exposed control group (*n* = 9) (Fig 8C and 8F; one-way ANOVA, *F*_2,17_ = 6.11, *P* = 0.01; posthoc comparison noise versus control, *P* < 0.01), and this too was prevented by dTT treatment (*n* = 5) (noise/dTT versus noise, *P* < 0.05; noise/dTT versus control, *P* = 0.54). The three groups were not significantly different in their mEPSC amplitudes (Fig 8C and 8G; *F*_2,17_ = 2.29, *P* = 0.13). These results confirm previous findings that NIHL results in increased excitatory and reduced inhibitory synaptic transmission, causing an excitatory-to-inhibitory synaptic imbalance, and suggest that this imbalance is largely mediated by noise-induced neuroinflammatory responses.

### Treatment with dTT alleviates noise-induced tinnitus measured with a conditioned lick suppression paradigm

**Fig 9:**
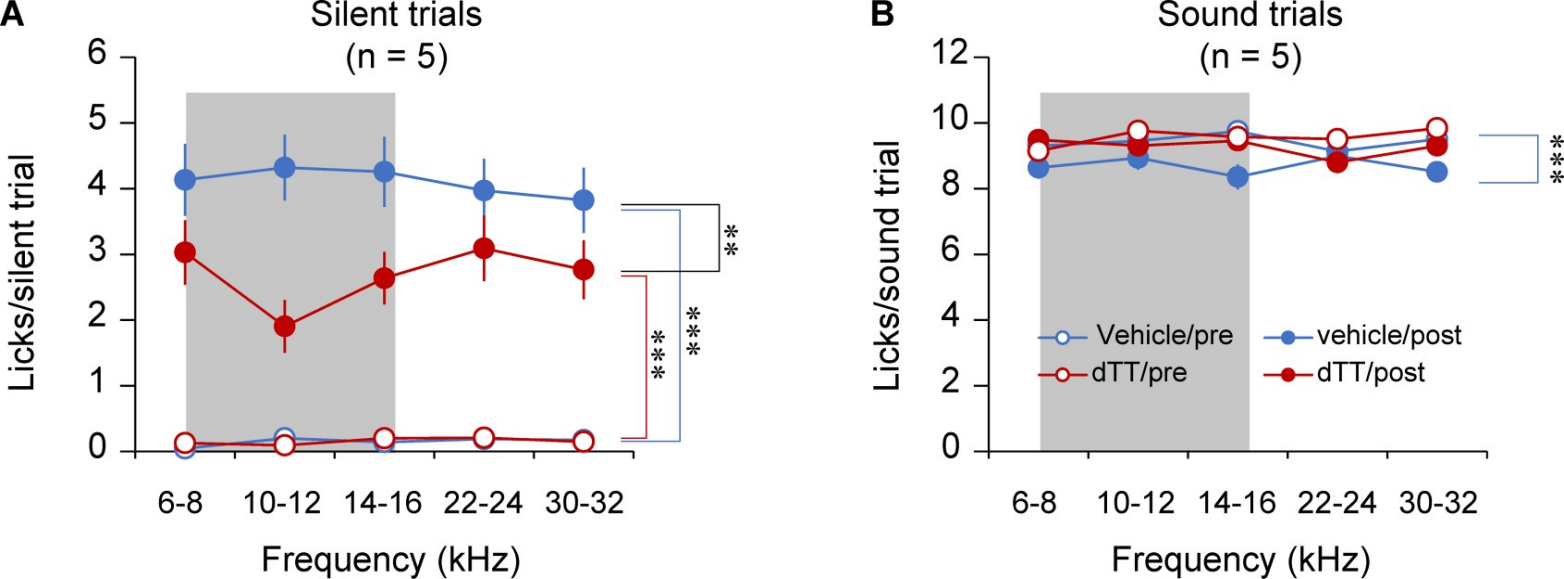
Treatment with dTT alleviates noise-induced tinnitus measured with a conditioned lick suppression paradigm. Licking rates during sound and silent trials were measured before and after noise exposure with dTT or vehicle injections. Both vehicle- and dTT-injected animals had similar baseline licking rates during silent and sound trials. Following noise exposure, all groups significantly increased their licking rates during silent trials, suggesting that they perceived tinnitus and increased licking in order to receive presumed water rewards. However, dTT-treated animals licked significantly less during silent trials compared to vehicles. This suggests that dTT suppressed tinnitus perception. Importantly, sound trial licking remained consistent following noise exposure and dTT or vehicle injections, indicating that manifestation and suppression of tinnitus-like behavior, or silent trial licking, was not due to overall changes in behavior. The shaded area represents the band of noise exposure (8–16 kHz). Data associated with this figure can be found in S1 Data. Error bars depict SEM. ** and *** indicate *P* < 0.01 and *P* < 0.001, respectively. dTT, 3,6′-dithiothalidomide.

To verify the role of neuroinflammation in noise-induced tinnitus, we used a different rodent model of tinnitus and our newly developed conditioning-based behavioral test [66]. Awake rats were binaurally exposed to loud band-limited noise to induce tinnitus. Following noise exposure, half of the rats received dTT and the other half received vehicle. All rats were tested with a conditioned lick suppression task, in which they were trained to lick for water when a narrowband sound was played and to suppress water licking when it was silent. Before the noise exposure, rats consistently licked during sound trials and successfully suppressed licking in silent trials (Fig 9; ANOVA comparing licks during sound trials versus during silent trials: dTT group, *F*_1,193_ = 1,796.414, *P* < 0.001; vehicle group, *F*_1,136_ = 690.958, *P* < 0.001). After noise exposure, the number of licks in sound trials decreased from 9.5 licks per trial before exposure to 9 licks per trial after exposure (see Fig 9; ANOVA comparing licks during sound trials before versus after noise exposure, *F*_1,204_ = 13.926, *P* < 0.001), whereas the licks in silent trials significantly increased for both dTT- and vehicle-treated groups (drug × exposure two-way measures ANOVA comparing licks in silent trials: dTT group, *F*_1,29_ = 67.12, *P* < 0.001; vehicle group, *F*_1,27_ = 124.77, *P* < 0.001), suggesting that the animals perceived phantom sounds (i.e., tinnitus). However, administration of dTT after noise exposure significantly reduced the number of licks in silence, suggesting that the drug alleviated tinnitus (ANOVA drug × exposure interaction, *F*_1,56_ = 9.57, *P* = 0.003).

## Discussion

In this study, we demonstrated a potential role of neuroinflammation in noise-induced tinnitus. We documented a series of neuroinflammatory events in the auditory cortex following noise exposure—a rapid bilateral increase of TNF-α mRNA levels, followed by a steady contralateral increase of TNF-α protein levels; a subsequent, predominantly contralateral change of microglia morphology; and an increased expression of additional proinflammatory cytokines IL-1β and IL-18. Microglial activation and proinflammatory cytokines—the two defining aspects of neuroinflammation—appear to depend on each other in noise-induced neuroinflammatory responses. Pharmacologically depleting microglia prevents noise-induced increases in TNF-α, whereas genetic deletion or pharmacological blockade of TNF-α prevents noise-induced changes in microglial morphology. This is consistent with existing literature showing that activated microglia release proinflammatory cytokines and that proinflammatory cytokines further activate microglia [22, 25, 28, 44]. Our results suggest that noise exposure can initiate such a self-sustained, positive feedback loop of neuroinflammatory responses. Furthermore, noise-induced neuroinflammatory responses could result in excitatory-to-inhibitory synaptic imbalance and cause behavioral/perceptual pathologies such as tinnitus.

Previous research reported that peripheral microglial activation and increased TNF-α expression occurred in the inner ear following noise trauma [21, 67–70]. In the central nervous system, neuroinflammatory responses have been shown in the cochlear nucleus, the first relay station along the central auditory pathway, following auditory nerve transection or noise exposure [21, 71–73]. Our results here extend previous findings by showing noise-induced neuroinflammation in the auditory cortex, the final destination along the central auditory pathway. Thus, it appears that hearing loss may cause neuroinflammation in the entire auditory pathway. However, the specific manifestations of neuroinflammation may be different amongst different auditory brain areas. For example, noise exposure causes massive up-regulation of microglial markers in the cochlear nucleus [19, 20] but only results in modest changes in microglial morphology in the auditory cortex (see Fig 2). The profound neuroinflammatory responses in the subcortical auditory pathway suggest that they may play an important role in noise-induced tinnitus and other pathologies. This is consistent with observations of putative neural correlates of tinnitus in those areas [74, 75]. Therefore, it is possible that the tinnitus induced by cortical TNF-α infusion (Fig 4) was weaker than that induced by noise exposure (Fig 3C) because of the lack of subcortical involvement.

We exposed the left ear of the mice to a continuous 8-kHz tone at 112–114 dB SPL, centered at 8 kHz for 2 hours, with the right ear protected. Although the left ear was deafened rapidly within 20 minutes, the right ear continued to be exposed to the noise at a relatively loud level for an extended period during the noise exposure (Fig 1A). Since activity of AI neurons is driven predominantly by the contralateral ear, left AI should have received more acoustic stimulation compared to the right AI during the noise exposure. Interestingly, we see a stronger increase in the TNF-α mRNA level in the left than right AI shortly after the noise exposure, suggesting the possibility that it was caused by prolonged noise-driven neural activity through the right ear. The subsequent and more pronounced increase in its protein level in the right AI indicates a post-transcriptional regulation of TNF-α expression, possibly by NIHL in the left ear.

Exposure to loud noise increases the expression of TNF-α and causes inflammatory responses in the cochlea [21, 67–70]. Blocking TNF-α by etanercept has been shown to prevent NIHL [76]. However, we observed a similar level of NIHL between dTT-treated and vehicle-treated mice. This difference could be due to differences in the drugs and experimental conditions (e.g., sound loudness of 113 dB versus 106 dB, exposure duration of 2 hours versus 30 minutes, or recording hours versus days after noise exposure). While high-frequency hearing loss of up to 60 dB has been reported in the TNF-α knockout mice [77], we observed much smaller threshold increases in auditory cortical responses [78] and ABRs. Furthermore, in the present study, ABR thresholds remained similar between wild-type and TNF-α knockout mice measured following noise exposure and behavioral tests (Fig 3E).

Several types of brain cells can synthesize TNF-α, including microglia, neurons, and astrocytes [21, 54]. We found that the rapid noise-induced increases in TNF-α mRNA levels depended mainly on microglia, as removal of a large proportion of the microglia by PLX3397 inhibited both the basal level and the noise-induced increase of TNF-α mRNA. This raises the question of what signals activate microglia to result in an increase in TNF-α expression. Microglia can be activated by a range of molecules produced by pathogens, neurons, and other microglial/immune cells. These include lipopolysaccharides, cytokines, neurotransmitters, proteases, and β-amyloid peptide. For example, microglia express glutamate and adenosine receptors [79, 80]. The sound-driven activity during the noise exposure could trigger enough release of glutamate and adenosine [81] as to activate microglia [79, 82]. On the other hand, neuronal activity can also suppress microglia, partly by activity-dependent release of neurotrophins [83]. This suppression might be weakened in AI if neuronal activity is reduced by NIHL. The stronger hearing loss in the exposed ear compared to the protected ear may explain the more pronounced increase in TNF-α protein levels and microglial activation in the contralateral AI.

In the present study, noise-induced microglial deramification required an increase in TNF-α expression. Both genetic knockout of the TNF-α gene and pharmacological blockade of the increase of TNF-α expression prevented noise-induced microglial deramification. This is in contrast to blast-induced microglial deramification, which was not blocked by the same TNF-α blocker [84]. It is possible that blast-induced damage to the brain tissue causes microglial deramification through alternative TNF-α–independent mechanisms.

NIHL has been shown to up-regulate glutamatergic synapses and down-regulate gamma-aminobutyric acid (GABA) synapses in the auditory pathway [3, 6–10]. These types of homeostatic plasticity are implicated in tinnitus and other hearing loss–related pathologies. However, their underlying mechanisms are not entirely clear. Our results suggest that glutamatergic and GABAergic synapses are synergistically regulated by TNF-α, strengthening and weakening glutamatergic and GABAergic synapses, respectively, which leads to a synaptic imbalance. Blocking the noise-induced increase in TNF-α expression prevented both excitatory and inhibitory synaptic changes and the synaptic imbalance. This is consistent with the previously demonstrated role of TNF-α in modulating membrane expression, phosphorylation, and subunit composition of α-amino-3-hydroxy-5-methyl-4-isoxazolepropionic acid receptor (AMPAR) and GABA-A receptors in vitro [13, 31, 60] and in vivo [85–88]. Thus, noise-induced neuroinflammation (i.e., microglial activation and TNF-α release) is likely a mechanism underlying the shift of excitatory-to-inhibitory synaptic balance following noise exposure and hearing loss.

Hearing loss is a risk factor for tinnitus [2]. We found that noise-induced neuroinflammation was correlated with impairment in gap detection but not in PPI, which has been considered by many as behavioral evidence of tinnitus in rodent models [53]. However, some have argued that, instead of tinnitus, impairment in gap detection may reflect a temporal processing deficit [89–95], a central auditory processing deficit that contributes to dyslexia [96–98]. To disambiguate the role of neuroinflammation in tinnitus, we performed a conditioning-based test for tinnitus [66]. We found that blocking TNF-α expression by dTT significantly reduced behavioral evidence of tinnitus in the conditioning-based test, confirming that neuroinflammation contributes to tinnitus etiology. The role of neuroinflammation in central auditory processing deficit remains to be evaluated. Further research in noise- and hearing loss–induced neuroinflammation may unveil new targets for treatment of tinnitus and other hearing loss–related disorders. It should be noted that neuroinflammatory responses defend the central nervous system from external and internal insults [22]. In addition, microglia and proinflammatory cytokines are involved in normal physiological functions [13, 27–33]. Although blocking neuroinflammatory responses prevented noise-induced tinnitus in the present study, its potential adverse effects need to be thoroughly investigated.

## Methods

### Ethics statement

All procedures have been approved by University of California–Berkeley, University of Arizona, and Wayne State University IACUCs.

### Animals

C57BL/6J and TNF-α knockout mice (B6.129S/SvEv-Gpi1-Tnf^*tm1Gkl*^) (8-week-old male, Jackson Laboratory, Bar Harbor, ME) and Sprague-Dawley rats (110–120-day-old males, Envigo Corporation, Huntingdon, United Kingdom) were used in the experiments.

### NIHL and ABR

Animals were anesthetized with ketamine (100 mg/kg, IP) and xylazine (10 mg/kg, IP) and maintained at 36.5°C with a homeothermic heating pad (Harvard Apparatus, Holliston, MA). Unilateral NIHL was induced by playing a continuous 112–114 dB SPL noise centered at 8 kHz through a custom-made piezoelectric earphone speaker to the left ear for 2 hours. The right ear was protected with sound attenuating clay. The sound level was measured with a Bruel and Kjaer 4135 condenser microphone (Nærum, Denmark).

Under ketamine anesthesia, ABR tests were performed to assess hearing thresholds before and at various time points after noise exposure. ABR signals were acquired using the BioSigRP software on a TDT RX5 Sys3 recording rig. Tone pips (3-ms full-cycle sine waves of various frequencies, with 2.5-ms Cos2-gated rise/fall time, at 5-dB intensity steps from 0 to 70 dB) were delivered to a single ear through a cannulated speaker at a rate of 19 times per second. The speaker was calibrated to have <3% harmonic distortion and flat output in the entire frequency range (Tucker-Davis Technologies SigCal32). Five hundred recordings were averaged for each frequency intensity pair. ABRs were visually identified by an experienced experimenter who was “blind” to the animals’ group identities. The lowest sound level that evoked discernable ABRs was determined at each testing frequency and recorded as the ABR threshold for that frequency.

### Drug administration

PLX3397 (Active Biochem LTD, Hong Kong) was applied in a suspension containing 2% dimethyl sulfoxide (DMSO), 1.5% Tween80, and 1% carboxymethyl cellulose at a concentration of 5 mg/mL. The naïve mice were given daily 30-mg/kg PLX3397 injections (IP) for 21 days and were randomly assigned into PLX/NIHL or PLX/naïve groups. The control group was treated with injections containing 2% DMSO, 1.5% Tween80, and 1% carboxymethyl cellulose at 6 mL/kg. The TNF-α mRNA level in AI was detected 12 hours after noise exposure.

dTT (ACME Bioscience) was prepared as a suspension in 1% carboxymethyl cellulose at a concentration of 50 mg/mL. The noise-exposed mice were randomly assigned into the NIHL/dTT group and were given daily 56-mg/kg dTT injections (IP). The other noise-exposed mice were assigned into the NIHL/vehicle group and treated with vehicle (1% carboxymethyl cellulose at 1.1 mL/kg) injection. The dTT and vehicle treatment started within 1 hour after noise or sham exposure and lasted for 5 days.

### Cortical infusion of recombinant TNF-α

Mice were anaesthetized with ketamine (100 mg/kg IP) and xylazine (10 mg/kg IP). An injection of recombinant TNF-α was placed stereotactically to the right auditory cortex through a burr hole that was made on the temporal ridge 1.75 mm anterior from the junction between the temporal ridge and the transverse suture. A glass micropipette filled with the recombinant mouse TNF-α (0.1 mg/mL dissolved in artificial cerebrospinal fluid and stabilized with 1 mg/mL mouse albumin fraction V) or mouse albumin fraction V (1 mg/in ACSF) solution was lowered down 500 μm from the pial surface, and 5 μL of solution was injected at 100 nL/min by pressure injection (Stoelting Quintessential Injector, Wood Dale, IL, USA). The micropipette was then retracted 250 μm, and an additional 5 μL of solution was injected. To minimize leaking, the micropipette was left in place for 8 minutes before being withdrawn. Animals were allowed 3 days for recovery before undergoing gap detection and PPI tests.

### Behavioral test of tinnitus with an acoustic startle reflex-based gap detection paradigm

During the testing session, a mouse was caged in a plastic container with a mesh lid. The container was placed on a piezoelectric sensor in a sound attenuation chamber. Sounds were played through an open field speaker (FOSTEX FT17H) fixed above the container. The gap detection task measured the acoustic startle response elicited by a brief white noise pulse and its suppression by a preceding silent gap embedded in a continuous background sound. Each trial started with a carrier pure tone (frequency pseudorandomly selected from 5, 7, 10, 14, 20, 28, and 45 kHz, all at 75 dB SPL) played for a duration of 10–20 s. In uncued trials, the carrier tone was followed by a startle stimulus—a 50-ms white noise burst at 102 dB SPL. In cued trials, a 50-ms silent gap in the background sound was introduced starting 100 ms before the onset of the loud noise burst. In each testing session, the animal underwent a total of 500 trials (50% cued and 50% uncued). After each session, we calculated the startle response ratio, which is defined as the average startle amplitude in the silent gap-cued trials divided by the average amplitude in the uncued trials. A lower startle response ratio indicates better detection of the silent gap. A startle response ratio of 1 suggests that the animal failed to detect the silent gap.

To assess an animal’s ability to hear a sound and perform an auditory task, animals underwent the PPI test before and after noise exposure. The test apparatus for the PPI task was identical to that of the gap detection. However, the test differed in that carrier tone was absent and a white noise burst was cued by a 50-ms pure tone pulse (frequency pseudorandomly selected from 5, 7, 10, 14, 20, 28, and 45 kHz, all at 75 dB SPL). In short, the PPI task tests an animal’s ability to detect a pure tone pulse in silence, while the gap detection task measures an animal’s ability to detect a silent gap in a continuous pure tone.

Gap detection and PPI performances were stabilized over two daily sessions each before data were collected. We compared individual animals’ performance before and after noise exposure or drug administration. An increase of gap ratio accompanied by i) normal ABR threshold for the intact ear and ii) normal PPI behavior were taken to indicate tinnitus. Because both the gap detection task and the PPI task require normal hearing and hearing sensitivity was highly variable across animals above 32 kHz, only trials with carrier frequencies between 5 and 20 kHz were included in the final analysis.

### Testing effects of dTT on tinnitus using a conditioned lick suppression behavioral paradigm

The behavioral test and experimental procedures were the same as previously reported [66]. Briefly, to induce tinnitus, awake adult male Sprague Dawley rats were binaurally exposed to an intense band-limited noise (8–16 kHz, 105 dB SPL, 2 hours) twice separated by 1 week. Sham-exposed rats underwent a similar procedure with the noise omitted. Before the noise exposure, animals were water deprived and trained to lick from a water spout only when a 2-kHz bandpass sound was played and to suppress water licking in silence. A partial reinforcement schedule was used, in which licks in sound trials were either rewarded or not rewarded with water, and licks in silent trials were punished or not punished with a mild foot-shock (0.25–0.75 mA and 1 s long; see [66] for details and rationale). Licks in sound trials were counted to quantify hearing and licking behavior, and licks in silent trials were counted to quantify tinnitus—lack of lick suppression in silence was taken as evidence of hearing phantom sounds. Performance in the task was examined before and after noise exposure.

### Quantitative real-time PCR

Mice were deeply anesthetized with isoflurane. The AI area tissue was collected and stored in 100 μL RIPA buffer (150 mM sodium chloride, 1.0% Triton X-100, 0.5% sodium deoxycholate, 0.1% sodium dodecyl sulphate [SDS], 50 mM Tris, pH 8.0) at −80°C. Tissues were homogenized in RIPA buffer by sonication. The total RNA was extracted by RNeasy Mini Kit (Qiagen, Valencia, CA). Immediately following extraction, the total RNA concentration and A260:A280 ratio of each sample were determined via NanoDrop 2000 (Thermo Scientific). The High Capacity cDNA Reverse Transcription kit (Thermo Fischer) was used to generate cDNA in a thermal cycler (ABI9700) for 2 hours at 37°C. Ten nanograms of cDNA were used in each reaction for real-time PCR using the CFX96 Real-Time PCR system (Bio-Rad Laboratories, Hercules, CA). Threshold cycle (Ct) values of the target genes were normalized to the endogenous control gene (ribosomal RNA, 18s). The primer sequences are listed as Table 1. Differential expression in experiment group samples relative to control samples was calculated using the comparative Ct method.

**Table 1:** Primers for RT-PCR.

Primer	Sequence
TNF-α–FW	CTGAACTTCGGGGTGATCGG
TNF-α–RV	GTGGTTTGCTACGACGTGGG
NLRP3-FW	ACCAGCCAGAGTGGAATGAC
NLRP3-RV	TGTAGCGACTGTTGAGGTCC
IL-1β–FW	TGCCACCTTTTGACAGTGATG
IL-1β–RV	TGGATGCTCTCATCAGGACAG
IL-18–FW	GACAGCCTGTGTTCGAGGAT
IL-18–RV	TGGATCCATTTCCTCAAAGG
18s-FW	GTAACCCGTTGAACCCCATT
18s-RV	CCATCCAATCGGTAGTAGCG

**Abbreviations:** FW, forward primer; RV, reverse primer.

### Immunostaining

Mice were transcardially perfused under deep anesthesia with ice-cold PBS, followed by 4% paraformaldehyde. Brains were removed and fixed in the same fixative overnight at 4°C, equilibrated in 30% sucrose, and embedded in Tissue-Tek (Sankura Finetek). Frozen coronal sections (16 μm) were collected on gelatinized glass slides. After air drying, sections were washed in PBS and penetrated with 0.1% Triton-X at room temperature for 10 minutes. The samples were blocked with Dako Serum-free blocking buffer (Dako) and incubated with primary anti-IBA1 Polyclonal antibody (ab107159, Abcam) overnight at 4°C. The secondary antibody conjugated with Alexa Fluor 568 (Invitrogen) were incubated for 1 hour at room temperature to enable fluorescent detection. After rinsing with PBS, the sections were mounted with fluorescence mounting medium (Dako) and viewed under the Olympus BX40 microscope with a digital microscope camera (C11440; Hamamatsu). Immunofluorescent images were systematically and randomly sampled from the AI area with the experimenters “blind” to the sample groups. Sample images were smoothed with a median filter and thresholded to remove background pixels according to the control images. The filter parameters and the threshold values were fixed across all groups for each experimental condition. All the images for the same experiment were taken on the same day using the same parameters. Analysis of the IBA1 staining was performed with the software Image J. Based on area coverage, the microglial cell size and the cell body size were determined manually. The soma-to-whole cell size ratio of the IBA1-stained cells was used to measure microglial deramification in the AI area [47, 48].

### Slice preparation and electrophysiological recordings

Mice were deeply anesthetized with isoflurane and then decapitated. The brain was extracted and immediately placed in an oxygenated (95% O_2_/5% CO_2_) external solution (87 mM NaCl, 2.5 mM KCl, 7 mM MgCl_2_, 25 mM D-glucose, 25 mM NaHCO_3_, 1.3 mM NaH_2_PO_4_, 0.5 mM CaCl_2_, and 75 mM Sucrose at pH 7.3). The brain was sectioned along the transverse plane into 300-μm slices with a vibrating microtome (Leica; VT1000S), and brain slices were stabilized in oxygenated external solution in a tissue chamber for 30 minutes at 32.5°C, followed by acclimation to room temperature (22.5°C) for at least 30 minutes before recordings. All recordings were performed at room temperature.

The chamber was continuously perfused with artificial cerebrospinal fluid (ACSF) (125 mM NaCl, 2.5 mM KCl, 1 mM MgCl_2_, 25 mM D-glucose, 25 mM NaHCO_3_, 1.3 mM NaH_2_PO_4_, and 2 mM CaCl_2_ at pH 7.3) at a rate of 2 mL/min. A fixed stage microscope (Olympus; BX51WI) equipped with differential interference contrast optics and a 40x water-immersion objective was used to visualize individual neurons in the auditory cortex.

Patch electrodes were fabricated from 1.5-mm diameter borosilicate glass micropipettes and had an impedance of 3–5 MΩ when back filled with the internal solutions for mIPSC and mEPSC measurement (135 mM Cs methanesulfonate, 10 mM CsCl, 10 mM Hepes, 0.2 mM EGTA, 4 mM ATP-Mg, 0.4 mM GTP-Na at pH 7.3, and 290 mOsm). In whole-cell configuration, the initial access resistance typically ranged from 15 to 23 MΩ and remained stable during the recording session.

Series resistance was also continuously monitored with a brief voltage pulse. Recordings were accepted when a cell had a resting membrane potential of at least −68 mV and a series resistance of 15–25 MΩ (<20% change during the recording session). mEPSCs were recorded from pyramidal neurons in Layers 2/3 in oxygenated ACSF solution containing 1 μM tetrodotoxin (TTX), with the cell held at −80 mV. mIPSCs were recorded in oxygenated ACSF solution containing 1 μM TTX, 10 μM 6-cyano-7-nitroquinoxaline-2,3-dione (CNQX), and 100 μM d-2-amino-5-phosphonovalerate (d-AP5), with the cell held at 0 mV. Signals were low-pass filtered at 2 kHz. All recordings were performed with Multiclamp 700B amplifier (Molecular Devices) and with pCLAMP10 software (Molecular Devices), and data were analyzed using MiniAnalysis software (Synaptosoft).

### Statistical analysis

Statistical analyses were conducted using MATLAB and SPSS (IBM). One-sample Kolmogorov–Smirnov tests suggest that our startle response ratio in gap detection and PPI tests did not significantly deviate from normal distributions [99]. ANOVAs were conducted to evaluate the statistical significance of the differences between groups or experimental procedures. For electrophysiological data, individual cells were used as the unit for analysis. The significance level was set at α = 5%. Data were presented as mean ± SEM unless otherwise stated.

## Supporting information

S1 Data Primary data set. This file contains individual numerical values used to generate figures in this manuscript.
(XLSX)

## Abbreviations

ABR: auditory brainstem response
AI: primary auditory cortex
AMPAR: α-amino-3-hydroxy-5-methyl-4-isoxazolepropionic acid receptor
CSF1R: colony-stimulating factor 1 receptor
dTT: 3,6′-dithiothalidomide
GABA: gamma-Aminobutyric acid
IBA1: ionized calcium binding adaptor molecule 1
IP: intraperitoneal
mEPSC: miniature excitatory synaptic current
mIPSC: miniature inhibitory synaptic current
NIHL: noise-induced hearing loss
NLRP3: nod-like receptor protein 3
PPI: prepulse inhibition
SPL: sound pressure level
TNF-α: tumor necrosis factor alpha
